# Myelin-Independent Therapeutic Potential of Canine Glial-Restricted Progenitors Transplanted in Mouse Model of Dysmyelinating Disease

**DOI:** 10.3390/cells10112968

**Published:** 2021-11-01

**Authors:** Luiza Stanaszek, Malgorzata Majchrzak, Katarzyna Drela, Piotr Rogujski, Joanna Sanford, Michal Fiedorowicz, Magdalena Gewartowska, Malgorzata Frontczak-Baniewicz, Piotr Walczak, Barbara Lukomska, Miroslaw Janowski

**Affiliations:** 1 NeuroRepair Department, Mossakowski Medical Research Institute, Polish Academy of Sciences, 02-106 Warsaw, Poland; lstanaszek@imdik.pan.pl (L.S.); zata.majchrzak@gmail.com (M.M.); progujski@imdik.pan.pl (P.R.); barbara.lukomska@imdik.pan.pl (B.L.); 2 Medical Research Agency, 02-106 Warsaw, Poland; kadrel@wp.pl; 3 Vetregen Laboratory and Stem Cell Bank for Animals, 04-687 Warsaw, Poland; joannasanford5@yahoo.com; 4 Small Animal Magnetic Resonance Imaging Laboratory, Mossakowski Medical Research Institute, Polish Academy of Sciences, 02-106 Warsaw, Poland; mfiedorowicz@imdik.pan.pl; 5 Electron Microscopy Platform, Mossakowski Medical Research Institute, Polish Academy of Sciences, 02-106 Warsaw, Poland; mgewartowska@imdik.pan.pl (M.G.); mbaniewicz@imdik.pan.pl (M.F.-B.); 6 Department of Diagnostic Radiology and Nuclear Medicine, Center for Advanced Imaging Research, University of Maryland Marlene and Stewart Greenebaum Comprehensive Cancer Center, University of Maryland, Baltimore, MD 21201, USA; pwalczak@som.umaryland.edu; 7 Department of Neurology and Neurosurgery, University of Warmia and Mazury, 10-082 Olsztyn, Poland

**Keywords:** GRPs, glial-restricted precursors, myelination, MRI, neurological disorders

## Abstract

Background: Dysfunction of glia contributes to the deterioration of the central nervous system in a wide array of neurological disorders, thus global replacement of glia is very attractive. Human glial-restricted precursors (hGRPs) transplanted intraventricularly into neonatal mice extensively migrated and rescued the lifespan in half of the studied mice, whereas mouse GRPs (mGRPs) presented no therapeutic benefit. We studied in the same experimental setting canine GRPs (cGRP) to determine whether their therapeutic potential falls between hGRPs and mGRPs. Additional motivation for the selection of cGRPs was a potential for use in veterinary medicine. Methods: cGRPs were extracted from the brain of dog fetuses. The cells were transplanted into the anterior or posterior aspect of the lateral ventricle (LV) of neonatal, immunodeficient, dysmyelinated mice (*Mbp*^shi^, *Rag2* KO; shiv/rag2). Outcome measures included early cell biodistribution, animal survival and myelination assessed with MRI, immunohistochemistry and electron microscopy. Results: Grafting of cGRP into posterior LV significantly extended animal survival, whereas no benefit was observed after anterior LV transplantation. In contrast, myelination of the corpus callosum was more prominent in anteriorly transplanted animals. Conclusions: The extended survival of animals after transplantation of cGRPs could be explained by the vicinity of the transplant near the brain stem.

## 1. Introduction

CNS diseases are becoming a major burden to society [1,2,3]. Glia are major components of the central nervous system [4], and contribute to a wide array of disorders including neurological [5,6] and psychiatric conditions [7,8]. Moreover, transplantation of glial progenitors has been shown to be therapeutic in small animal models of a variety of brain diseases such as Pelizeaus-Merzbacher disease (PMD) [9], amyotrophic lateral sclerosis (ALS) [10], Huntington’s disease [11], spinal cord injury [12] as well as in vitro models including neonatal hypoxia-ischemia [13]. However, the transplantation of myelin-producing progenitor cells in patients with PMD was largely ineffective. The closer analysis of clinical data revealed possible myelination only at a narrow area of the implantation site [14,15]. Indeed, we have recently shown in a mouse model of dysmyelination (*Mbp*^shi^; shiv, which was also immunodeficient to avoid graft rejection; rag2 knockout (KO)) that a robust therapeutic effect of human glial-restricted precursors (hGRPs) correlates well with their distribution throughout the entire mouse brain, whereas mouse GRPs (mGRPs) characterized by limited migration, failed to provide any therapeutic effect [16]. Interestingly, the mGRPs differentiated towards mature oligodendrocytes and myelinated much faster than hGRPs, thus overall, we have observed a radically different behavior of GRPs depending on the species they come from. We hypothesized there are at least two reasons for such behaviors: (1) the GRPs execute their own intrinsic developmental program (IDP), which is closely linked with the size of the brain and species-appropriate dynamics of myelination, more particularly hGRPs migrate far and myelinate late, and oppositely mGRPs migrate short and myelinate early, and (2) due to a human-mouse mismatch, xenografted hGRPs cannot fully respond to the host cues driving the maturation of oligodendrocytes, allowing them to remain longer in a highly migratory undifferentiated state and extensively migrate.

To test our hypotheses, we selected dogs as a species with a large brain to derive GRPs. If IDP would be a major driver of hGRP performance, we would expect that GRPs derived from dogs will perform somewhat between mGRP and hGRP in terms of migration and myelination. In contrast, if environmental factors such as the capability of transplanted cells to read the host clues would have a dominant role, then GRPs derived from large animals would migrate equally well as hGRP after transplantation to dysmyelinated mice, which would subsequently translate to their widespread benefit equal to observed in case of hGRPs. Even though allografting of mGRPs in our previous study was disappointing, it was important to study the performance of GRPs derived from other large brains, to fully understand the mechanisms governing engraftment and myelination. Such data will help maximize the success and predictability of clinical translation. Here, we used shiverer mice again due to the robust readouts they offer, such as lifespan at about six months, perfectly suited to reveal therapeutic effects of GRPs, as well as *Mbp* mutation rendering endogenous myelin undetectable by antibodies to MBP, which guarantees to ascribe any positive immune staining against MBP to the effect of the activity of transplanted cells. The lack of compact myelin in shiverer mice is another advantage as it allows to determine the axon myelination by transplanted cells using MRI and electron microscopy.

The selection of a dog as a large animal GRP donor has another very practical advantage. There is a growing population of dogs as companion animals, with neurological health issues requiring attention. One of the canine illnesses that resembles ALS is degenerative myelopathy (DM). DM affects mostly adult dogs and may be characterized by the loss of upper motor neurons and degeneration of nerve fibers. Moreover, canine degenerative myelopathy is reminiscent of ALS in terms of histopathological changes, clinical outcomes and mutations in genes such as SOD1 [17,18]. This creates a solid market and demand for the application of canine GRPs (cGRPs) in veterinary medicine. These therapeutic indications in veterinary medicine have important translational implications as many canine diseases closely resemble human diseases with an example of degenerative myelopathy being an excellent model of ALS [18,19]. cGRPs can also be relatively easily obtained in large quantities. In summary, transplantation of cGRP in a mouse model of dysmyelination was performed to get a closer insight into the reason behind a radically different outcome of transplantation of hGRP vs. mGRP, as well as their potential applicability in veterinary medicine.

## 2. Materials and Methods

### 2.1. Experimental Design

The main goal of this project was the assessment of the therapeutic capacity of cGRPs transplanted into immunodeficient, dysmyelinated mice. The study is composed of one independent variable and five dependent variables (endpoints). The transplantation site is an independent variable, and it is composed mainly of three levels: (1) anterior transplantation (A-transplanted at the level of bregma focused on targeting of the forebrain), (2) posterior transplantation (P-transplanted at the level of lambda focused on targeting of midbrain and hindbrain), (3) negative control (mutant mice without transplantation). Some endpoints included the fourth level: positive control composed of wild-type mice without defect in myelin. The dependent variables include (1) animal life span, (2) myelination detected by magnetic resonance imaging (MRI), (3) myelination detected by immunohistochemistry (IHC), (4) myelination detected by electron microscopy (EM), (5) early bio-distribution of cGRPs.

Animal life span was measured by recording the time animals died. Shiverer mice die unexpectedly; therefore, it is difficult to collect good quality tissue. Furthermore, each endpoint measure requires different tissue preservation, thus separate cohorts of animals had to be used for the assessment of postmortem endpoints such as IHC, EM and assessment of biodistribution.

The animals were sacrificed at three time points: 18, 31 and 40 weeks after neonatal transplantation. Post-mortem assessment of myelination was performed only in anteriorly transplanted animals, as MRI reported no myelination in posteriorly transplanted animals.

Animals for longitudinal in vivo MRI were randomly recruited from the cohort devoted to the assessment of life span and IHC.

For assessment of early bio-distribution, cGRPs were fluorescently labeled with Dil and followed postmortem at three time points: 1, 3 and 28 days. In total, there were 139 mice used in the study and the animal allocation to experimental and control groups is presented in Table 1.

All the procedures were conducted with appropriate approval from the Ethical Committee (IV Local Committee in Warsaw, 48/2013, 240/2017, 259/2017).

### 2.2. Procurement of cGRPs and Preparation for Transplantation

The procedure of cGRP isolation followed the methodology for mGRP derivation [20]. Briefly, cGRPs were derived from mid-gestation (E 32–37) dogs. Isolated brains and spinal cords from canine fetuses were sectioned into small fragments and incubated with TrypLE^TM^ Express (Thermo Fisher Scientific, Waltham, MA, USA) with the addition of 10 mg/mL of DNase-1 (Sigma-Aldrich, St. Louis, MO, USA) in RT for 10–12 min then the tissues were triturated and incubated for 10 min in 37 °C. TrypLE^TM^ was inactivated through the addition of 5 mL of complete GRP medium and the cell suspension was spun down (300× *g*, RT, 5 min), the supernatant was discarded, the pellet was suspended in GRP medium with 10 mg/mL of DNase and incubated for another 10 min in 37 °C followed by cell trituration and spinning down (300× *g*, RT, 5 min). Then, the pellet was re-suspended in GRP medium [DMEM/F12 (Thermo Fisher Scientific, Waltham, MA, USA), N-2 Supplement (Thermo Fisher Scientific, Waltham, MA, USA), B-27 Supplement (Thermo Fisher Scientific, Waltham, MA, USA), bovine serum albumin 0.1% (Sigma-Aldrich, St. Louis, MO, USA), heparin 1 µg/mL (Sigma-Aldrich, St. Louis, MO, USA), Penicillin-Streptomycin (Thermo Fisher Scientific, Waltham, MA, USA) with the addition of 20 ng/mL bFGF-2 (Takara, Kusatsu, Japan)], obtained cells were plated on poly-L-lysine (Sigma-Aldrich, St. Louis, MO, USA) and laminin (Thermo Fisher Scientific, Waltham, MA, USA) coated flasks and cultured in standard cell culture conditions (37 °C, 5% CO_2_ concentration) for two passages. GRPs were then harvested, cryopreserved in ATCC medium (ATCC, Manassas, VA, USA) and stored in liquid nitrogen. Before transplantation, cGRPs were thawed, counted and suspended in sterile saline. Labeling of cGRPs with lipophilic dye–DiL (Thermo Fisher Scientific, Waltham, MA, USA) has been performed according to the manufacturer’s instructions for their postmortem identification in short-term experiments to assess their early intracerebral bio-distribution.

### 2.3. Immunocytochemistry

In order to characterize the cellular profile of transplanted cGRPs cells were subjected to immunocytochemistry (ICC). First, cGRPs in passage 2 were thawed from liquid nitrogen and seeded on poly-L-lysine and laminin-coated coverslips. Second, cGRPs were fixed with 4% paraformaldehyde solution for 20 min and washed 3 times in PBS. Next, blocking solution (10% natural goat serum, 5% bovine serum albumin, 0.25% Triton X-100) was applied for 1 h at RT. The following primary antibodies were used overnight at 4 °C: A2B5 (1:200) (MAB312R, Merck KGaA, Darmstadt, Germany), PDGFRα (1:200) (sc-338, Santa Cruz Biotechnology, Dallas, TX, USA), GalC (1:200) (MAB342, Merck KGaA, Darmstadt, Germany), GFAP (1:200) (Z0334, DAKO, Jena, Germany), NG2 (1:200) (AB5320, Merck KGaA, Darmstadt, Germany), Ki67 (1:10) (PA0118, Leica Biosystems, Wetzlar, Germany), Olig1 (1:500) (AB15620, Merck KGaA, Darmstadt, Germany), Olig2 (1:500) (ABN899, Merck KGaA, Darmstadt, Germany), MBP (1:200) (MAB382, Merck KGaA, Darmstadt, Germany), CNPase (1:200) (AB9342, Merck KGaA, Darmstadt, Germany), O4 (1:200) (MAB345, Merck KGaA, Darmstadt, Germany). The next day, cells were washed 3 times in PBS, and appropriate secondary antibodies conjugated to Alexa fluorochromes were used for 1 h at RT: goat anti-mouse 488, goat anti-rabbit 546, goat anti-chicken 488, goat anti-chicken 546 (all in 1:500) (Thermo Fisher Scientific, Waltham, MA, USA). Nuclei were counterstained with 5 μg/mL DAPI (Thermo Fisher Scientific, Waltham, MA, USA) for 5 min at RT. Coverslips were embedded on microscope slides using Dako Fluorescence Mounting Medium (DAKO, Jena, Germany). Cells were imaged by Axio Observer LSM 780 (Carl Zeiss, Jena, Germany).

### 2.4. Shiv/rag2 Mice

In order to investigate the therapeutic activity of cGRPs we used double mutant immunodeficient, dysmyelinated shiverer mice (*Mbp*^shi^, *Rag2*KO) both males and females as previously described by us [16]. Animals were obtained through the cross-breeding of shiverer mice (C3HeB/FeJ-shiverer, The Jackson Laboratory, Bar Harbor, ME, USA) and *Rag2* KO mice (B6(Cg)-*Rag2*tm1.1Cgn/J, The Jackson Laboratory, Bar Harbor, ME, USA). For clarity, we are using “shiv/rag2” as simplified nomenclature for double mutants. Shiverer mice are characterized by a recessive autosomal mutation in both alleles of the *Mbp* (myelin basic protein) gene resulting in protein truncation and subsequent dysmyelination. A characteristic feature of shiverer mice is trembling during locomotion, occasional seizures and lifespan shortened to around 200 days. In turn *Rag2* knockout results in defects in T and B cell development and thus impaired adaptive immunological response. Mice are genetically stable and some of the randomly chosen animals were genotyped in order to confirm the mutation in the *Rag2* gene. Homozygous mutation of the *Mbp* gene is phenotypically visible through trembling. Due to immunodeficiency mice were bred and maintained in a special pathogen-free (SPF) environment in the Laboratory of Genetically Modified Animals at Mossakowski Medical Research Institute, PAS.

### 2.5. cGRP Transplantation

Mouse pups (P 2–3) were cryo-anesthetized and placed on ice in stereotaxic apparatus equipped with a mouse adaptor. cGRPs (total 2 × 10^5^ cells in 4 uL of saline) were transplanted intraventricularly into each hemisphere using stereotaxic coordinates: AP:0.6, ML:1.0/−1.0 and DV:0.8 from bregma (anteriorly (A)-transplanted) or AP:0.8, ML:1.0/−1.0, DV:0.8 from lambda (Posteriorly (P)-transplanted). Control mice did not receive cGRP transplantation. Anteriorly and posteriorly transplanted mice pups were randomly assigned to A- or P-injection site group and performed diversely during the same or different surgery sessions. After transplantation, the pups were removed from the stereotaxic apparatus, their vital functions were restored and they were returned to the cage with their mother.

### 2.6. Immunohistochemistry

For immunohistochemical studies, the experimental and control mice were sacrificed after deep anesthesia with a mixture of 100 mg/kg ketamine and 1 mg/kg medetomidine i.p. injection. Then, the animals were perfused intra-cardially with 4% paraformaldehyde (PFA). The brains and spinal cords were removed, post-fixed overnight in 4% PFA and incubated in 20% saccharose until fully saturated and cryopreserved with dry ice and stored in −80 °C until cryo-cut into 20 µm sections for fluorescent immunohistochemistry. Briefly, tissue sections were rinsed in PBS and then incubated in 10% goat serum in PBS containing 0.25% Triton X-100 and 0.1% BSA for 60 min in room temperature (RT). Next, the sections were washed with PBS and incubated with primary antibodies (60 min, RT). The following antibodies (final dilution and source) were used for brain and spinal cord tissue staining: rat anti-MBP (myelin basic protein) monoclonal antibody (1:200, Merck KGaA, Darmstadt, Germany) to detect myelin protein; anti-GFAP (glial fibrillary acidic protein, 1:500, DAKO, Jena, Germany) polyclonal antibody to display astrocytes and neural progenitors. Then, tissue sections underwent the washing procedure, and the primary antibodies were visualized by applying appropriate secondary antibodies: goat anti-rat or goat anti-rabbit Alexa Fluor 488 or 546 (1:500, Thermo Fisher Scientific, Waltham, MA, USA) for 60 min at RT and in the dark. Nuclei were counterstained with the fluorescent dye 5 μM Hoechst 33258 (Sigma-Aldrich, St. Louis, MO, USA). Images of immunostained tissues were acquired using a confocal laser scanning microscope (LSM 780, Carl Zeiss, Jena, Germany) and Cell Observer SD (Carl Zeiss, Jena, Germany) using a 20× or 40× objectives. A helium-neon laser (543 nm) was utilized in the excitation of Alexa Fluor 546, whereas an argon laser (488 nm) was applied in the excitation of FITC. ZEN software was used for quantitative analysis of immunoreactivity in all sections. Five animals per group were analyzed. Images from two sections per animal were acquired, and the number of positively stained cells was counted as well as fluorescence intensity was measured. The analysis was performed in the Laboratory of Advanced Microscopy Techniques, Mossakowski Medical Research Institute, PAS.

### 2.7. Transmission Electron Microscopy (TEM) Analysis

The myelin visualization in the brain and spinal cord of A-transplanted animals was performed in mice sacrificed at two points: 18 weeks and 31 weeks post cGRP transplantation. Non-transplanted, dysmyelinated *Rag2* KO animals served as a negative control and healthy *Rag2* KO mice served as a positive control. Before isolation of regions of interest, the experimental and control animals were anesthetized with a mixture of 100 mg/kg ketamine and 1 mg/kg medetomidine and perfused intra-cardially with 0.9% NaCl in 0.01 M sodium-potassium phosphate buffer (pH 7.4), followed by 2% paraformaldehyde and 2.5% glutaraldehyde in 0.1 M cacodylate buffer (pH 7.4) infusion. Tissue samples collected from the corpus callosum and white matter of spinal cords were fixed in the above-mentioned ice-cold fixative solution and post-fixed in 1% OsO_4_ solution. After dehydration in the ethanol gradient, the tissue samples were embedded in epoxy resin (Epon 812) (Sigma-Aldrich, St. Louis, MO, USA). Thin sections of 50 nm were stained with 9% uranyl acetate and lead nitrate. Images were acquired using JEM-1200 EX (Jeol, Tokyo, Japan) transmission electron microscope equipped with MORADA camera and iTEM 1233 software. TEM analysis was performed in the Electron Microscopy Platform, Mossakowski Medical Research Institute, PAS.

### 2.8. MRI Analysis

MR imaging was performed 18, 32 or 40 weeks after cell transplantation. Mice were anesthetized with isoflurane (1.5–2% in oxygen) and positioned headfirst, prone in the MR-compatible water-heated bed. Body temperature and respiration rate were monitored throughout the study with MR-compatible probes (SA Instruments, Stony Brook, NY, USA). 7T MRI scanner (BioSpec 70/30 USR, Bruker, Ettlingen, Germany) equipped with transmitting cylindrical radiofrequency coil (8.6 cm inner diameter, Bruker, Ettlingen, Germany) and a mouse brain dedicated receive-only array surface coil (2x2 elements, Bruker, Ettlingen, Germany) were used. The structural imaging protocol was performed as we described previously [21]. Briefly, we used T2-weighted TurboRARE sequence (TR = 7000 ms; TEeff = 15 ms; RARE factor = 4; NA = 4; field of view, FOV = 22 mm × 22 mm; spatial resolution = 86 µm × 86 µm × 350 µm; 42 slices, no gap; scan time~23 min). Measurements of signal intensity in the corpus callosum were performed in Fiji software. Briefly, the same size of corpus callosum midsections was outlined on T2 MRI images of a similar coronal slice. The intensity of the signal was measured and normalized to the signal intensity of the cortex for identical size ROI. Imaging was performed in Small Animal Magnetic Resonance Laboratory, Mossakowski Medical Research Institute, PAS.

### 2.9. Statistical Analysis

The least means squares incorporated into PROC MIXED (SAS 9.4) (Cary, NC, USA) have been used to determine the differences in continuous variables between the groups for the MRI endpoint. The dot plot was employed for their graphical presentation. The chi-square test has been used to calculate significance in the frequency distribution table, which served to quantify differences in cell bio-distribution endpoint. The log-rank (Mantel–Cox) test was used to calculate differences in life span between groups. Kaplan–Meier curves to present animals’ survival were drawn using Graph Pad Prism 7.04 software (San Diego, CA, USA).

## 3. Results

### 3.1. Cellular Profile of cGRPs

**Figure 1:**
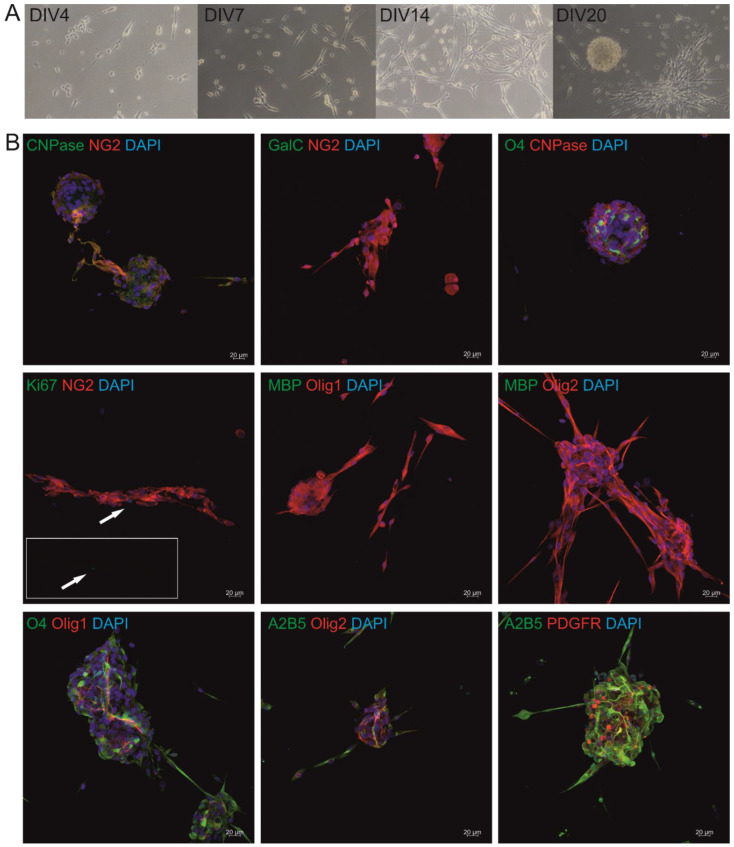
Images presenting cGRPs in culture. (**A**) transmitted light images of cGRPs on the p2 captured from the DIV4 until DIV20. (**B**) Immunocytochemical analysis confirming the phenotype of the cells. The cGRPs present CNPase, O4, NG2, Olig1 and 2, A2B5 and PDGFR antigens and are negative for MBP and GALC. Ki67 proliferation marker was visible in 17% of cells.

After the isolation, cells were propagated until passage 2–3 and froze. Throughout the cell culture, the morphology and behavior of the cells were analyzed through observation in the transmitted light microscope. In the beginning, cells were the mixture of the neural cells; however, culturing in a GRP-dedicated medium led to the selection of the cells with the glial progenitor phenotype. Within the first day of culture, the cells created a monolayer, however, after a longer period in culture cGRPs tended to form characteristic spheres, attached to the surface. After further time in culture, cells were spreading out of the spheres and created contacts with other cells/spheres (Figure 1A). The cells were also subjected to immunocytochemical analysis. On the passage, 2 cells presented all the markers representative for oligodendrocyte precursors such as CNPase, O4, NG2, Olig1 and 2, A2B5 and PDGFR antigens and are negative for MBP and GALC. However, at this stage, markers characteristic for mature oligodendrocytes (MBP) were not present (Figure 1). Moreover, cells were positive for GFAP marker (data not shown) and 17% of the cells presented the Ki67 positive signal. We performed quantification of the cellular profiles of cells grown in vitro: ~100% of cells were positive for NG2, ~100% for OLIG1, and ~100% for OLIG2. No MBP+ cells were identified within the culture (Figure 1B).

### 3.2. Survival Analysis of shiv/rag2 Mice Transplanted with cGRPs

**Figure 2:**
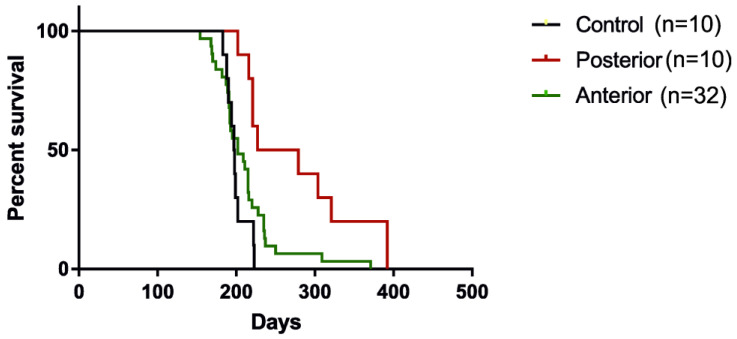
Animal survival (shiv/rag2 mice) presented as a Kaplan–Meier curve. P-transplanted mice at the level of lambda survive longer than A-transplanted mice at the level of bregma and non-transplanted mice.

The median survival of control mice (shiverer, *Mbp*^shi^, *Rag* KO; shiv/rag2) was 197.5 days, and the median survival of A-transplanted mice was extended only to 202 days (*p* = NS), whereas mice who received P-transplants revealed a significant extension of median survival to 253 days (*p =* 0.0005) (Figure 2). More descriptively, there were only 10% of A-transplanted mice which survived longer than 10% of the time of the longest-surviving non-transplanted mouse (around 20 days), whereas the same was observed in 50% of P-transplanted mice. The statistical significance was performed using the Mantel-Cox test, where *p*-value = 0.0025.

### 3.3. Analysis of the Early Distribution of cGRPs within the Brain after Their Transplantation into Two Different Transplantation Sites

**Figure 3:**
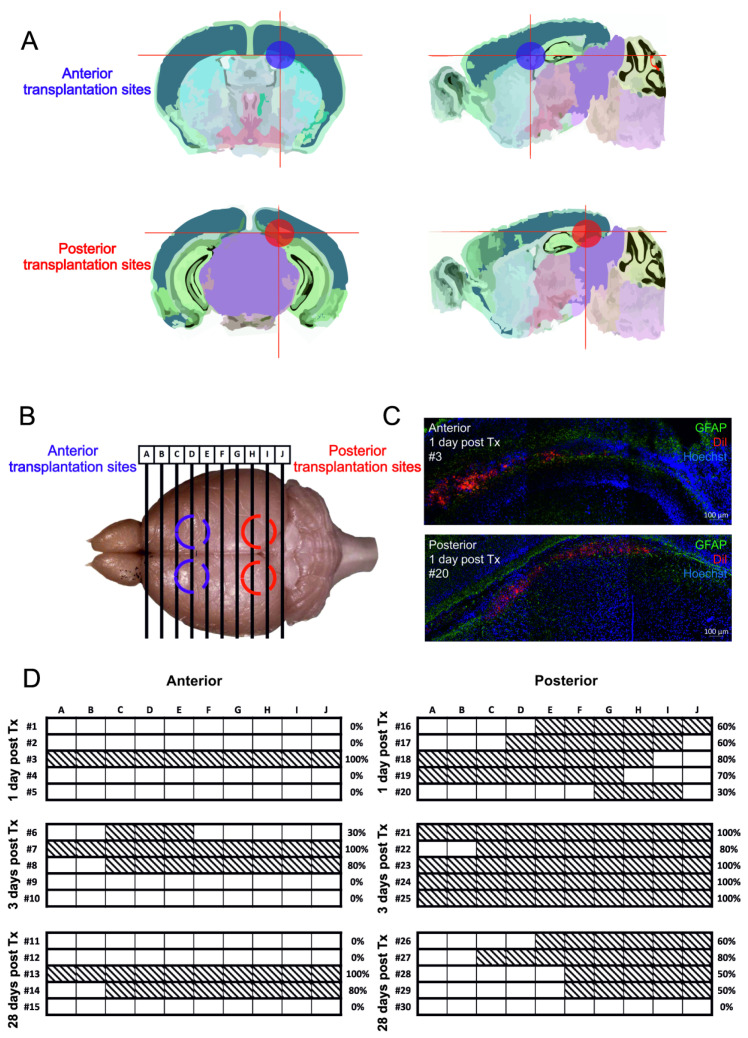
Cerebral biodistribution of cGRPs transplanted to two different injection sites: anterior and posterior. (**A**)—schematic representation of transplanted cells localization depending on transplantation site (schematics are adapted from mouse Allen brain atlas), (**B**)—schematic presentation of analyzed brain areas, (**C**)—representative images of coronal brain slices stained for GFAP (green) and Hoechst (blue) with Dil-stained cells visible in red. (**D**)—schematic representation of cGRPs bio-distribution across the brain areas. Slices were analyzed 1, 3 or 28 days after transplantation. Each section within a row, indicated by letters A–J, reflects a 60 µm long brain area, with a 240 µm step between subsections. Patterned sections reflect Dil-positive areas, corresponding to cGRPs localization. Each hashtag #1–#30 represents one animal. Percentage reflects overall Dil bio-distribution across analyzed brain slices.

The injection site in the A-transplantation group corresponds to anterior horns of the lateral ventricle, whereas for the P-transplantation group, the cells were infused to the area of ventricle lining hippocampus and neighboring midbrain (Figure 3A,B). In the A-transplantation group, cGRPs were detected in 6 out of 15 animals, whereas for the P-transplantation group in 14 out of 15 animals (chi-square = 9.6, *p* < 0.01). Mapping bio-distribution of cGRPs confirmed their presence across the entire ventricular system in both groups of animals, although as expected more cells were present around their transplantation sites (Figure 3C,D; Supplementary Figure S1).

### 3.4. MRI Analysis of Myelination after cGRPs Transplantation in shiv/rag2 Mice

**Figure 4:**
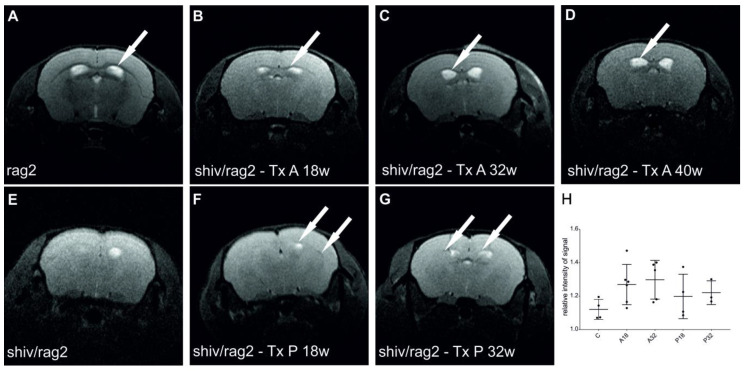
MR imaging of myelination in the corpus callosum (cc) of shiv/rag2 mice. Hypo-intensity on MR images reflects myelinated axons in cc (arrows). MR scanning was performed at different times post-transplantation: 18 weeks (**B**,**F**), 32 weeks (**C**,**G**) and 40 weeks (**D**). Positive myelination control—200-day old *Rag2* KO mice is shown on (**A**). I—represents negative myelination control—200 days old non-transplanted shiv/rag2 mice. (**B**–**D**) denotes MR images of mice transplanted with cGRPs on A-transplantation site (A-AP:0.6, ML:1.0/−1.0, DV:0.8 from bregma). MR images of mice transplanted with cGRPs on the posterior transplantation site are presented on (**F**,**G**). (P-AP:0.8, ML:1.0/−1.0, DV:0.8 from lambda). (**H**)—Dot graph representing relative hypointensity signal in the corpus callosum of control animals and mice transplanted anteriorly and posteriorly at 18 and 32 weeks.

MRI analysis revealed strong T2 hypo-intensity in the corpus callosum in *Rag2* KO animals (Figure 4A), and no hypo-intensity in this area in shiv/rag2 mice (Figure 4E), confirming it as an excellent surrogate imaging marker of myelination. T2 hypointensity in the corpus callosum was also observed in both experimental (transplanted) groups (Figure 4B–D,F,G), though was much weaker than in positive controls (*Rag2* KO). Interestingly, the T2 hypo-intensity was more prominent in A-transplanted mice and the area of myelination for both groups was rather limited and there was no expansion of that area over time beyond the earliest MRI time point at 18 weeks (Figure 4B–D). As expected, there was a variability of T2 hypo-intensity among all animals. The analysis of MRI signal hypo-intensity revealed that myelination was pronounced in 18-week and 32-week animals transplanted in the anterior site in comparison with the non-transplanted group (*p =* 0.0486; *p =* 0.0262, respectively). Myelination of the corpus callosum of posteriorly transplanted animals was not significantly different from control animals (Figure 4H).

### 3.5. Immunohistochemical Analysis of Myelin after cGRPs Transplantation in shiv/rag2 Mice

**Figure 5:**
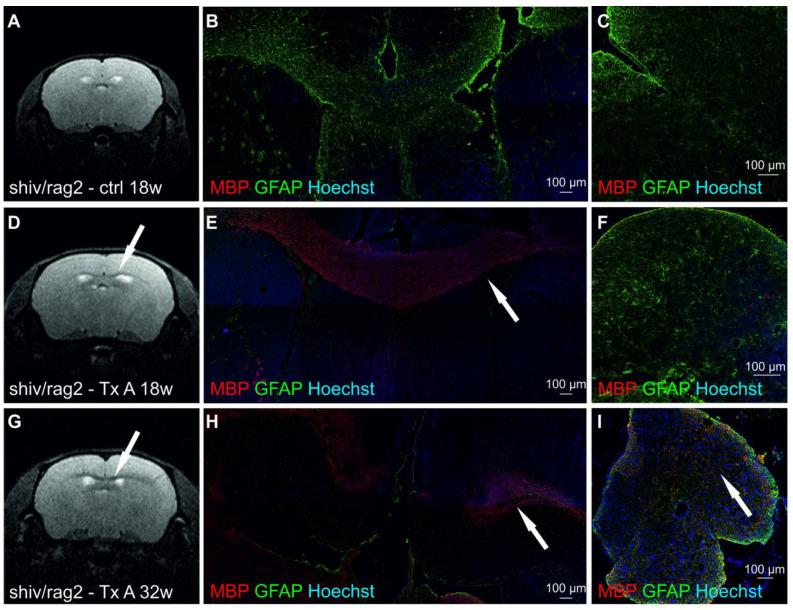
MR images and immunohistochemical staining presenting myelination in the corpus callosum and spinal cord of mice transplanted with cGRPs and control animals. (**A**–**C**)—presents absence of myelination in cc on MR image (**A**) and immunohistochemical staining of control brain (**B**) and spinal cord (**C**). D–I represent myelinating processes (arrows) in cGRPs transplanted mice 18 weeks post-transplantation (**D**–**F**) and 32 weeks after transplantation (**G**–**I**).

Immunohistochemical analysis performed in our studies confirmed that there was no MBP staining in control, non-transplanted shiv/rag2 mice (Figure 5A–C). In contrast, positive MBP staining was observed in the corpus callosum of examined transplanted shiv/rag2 mice at 18 weeks (Figure 5E) and the 32-week time point (Figure 5H), whereas typically, no positive staining was observed in the spinal cord (Figure 5F), except one A-transplanted animal in which robust myelination was also observed in the spinal cord (Figure 5I).

### 3.6. TEM Analysis of Axon Myelination in cGRP Transplanted shiv/rag2 Mice

**Figure 6:**
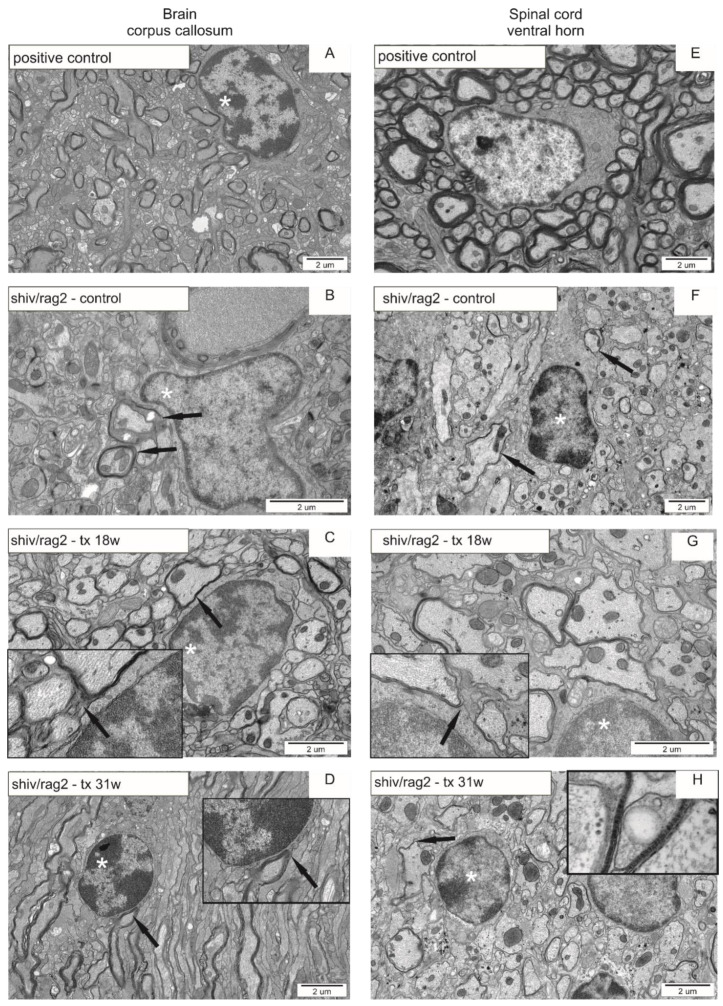
TEM pictures visualizing myelinated axons in CNS of control and GRP transplanted shiv/rag2 mice. The left panel represents corpus callosum whereas the right panel spinal cords of *Rag2* KO myelinated axons with visible compact myelin (**A**,**E**); axons without compact myelin (arrows) in negative control—shiv/rag2 mice without transplantation (**B**,**F**); shiv/rag2 mice 18 weeks (**C**,**G**) and 31 weeks (**D**,**H**) post-transplantation with visible compact myelin (arrows) around some of the axons. Asterisks indicate oligodendrocytes and insets represent an enlargement of the area of interest. Inset on (**H**) represents compact, mature myelin around the axon in the spinal cord.

In *Rag2* KO mice (positive control), as expected, there was a compact myelin structure in the corpus callosum and spinal cord (Figure 6A,E). There was no compact myelin observed in the brain and spinal cord of shiv/rag2 mice, except for single axons (Figure 6B,F). cGRP transplanted shiv/rag2 mice revealed groups of axons surrounded by compact myelin in the corpus callosum at 18 weeks (Figure 6C) and 31 weeks (Figure 6D). Moreover, actively myelinating oligodendrocytes were visible in the brains and spinal cords of transplanted mice. Proper, compact myelin around groups of axons (in contrast to single axons in the non-transplanted group) was visible in all animals of the transplanted group; however, there were visible differences in numbers of properly myelinated axons in one experimental group. Compact myelin was also observed in the spinal cord of some transplanted shiv/rag2 mice (Figure 6G,H).

## 4. Discussion

Our study revealed several surprising findings, which require extensive discussion in the context of existing literature. First of all, we have shown that myelin repair of corpus callosum does not go in pair with benefit in animal survival. The mice transplanted to the anterior part of the ventricular system did not show any meaningful extension of survival despite the robust myelination of the corpus callosum. In contrast, the animals transplanted to the posterior part of the ventricular system survived significantly longer, whereas the myelination of the corpus callosum was negligible. Moreover, the analysis of early cGRP biodistribution detected the cells in nearly all posteriorly transplanted animals, but cGRPs were found in only a small number of anteriorly transplanted animals, whereas assessment of myelination revealed compact myelin in all transplanted animals and none of the non-transplanted animals; there was high inter animal variability in the level of compact myelin formation.

The cGRPs myelinated early and robustly, as shown by MRI and postmortem analysis, but anteriorly transplanted animals did not show any survival benefit, which puts cGRPs in pairs with mGRPs, and not with hGRPs [16]. Interestingly, the same cGRPs transplanted more posteriorly significantly extended animal survival, which may be linked to the closer vicinity of the brain stem and might be a more essential brain structure for animal survival. However, the extension of animal survival by cGRP posterior transplantation was still less pronounced than by hGRPs in our previous study [16]. Therefore, we hypothesize that the behavior of transplanted GRPs might depend on IDP that is driven by a species mismatch between donor and host. It might be that due to genetic predispositions of cells isolated from particular species including its size, the transplanted cells might be naturally predisposed to behave adequately for the species origin.

The other possible explanation for differences in myelination, differentiation of transplanted cells as well as their survival is the particular niche in which the cells were transplanted. It is known that the environment of the host is of a great influence due to the vicinity of other resident cells that create a specific environment and release the factors that drive transplanted cells to behave according to the particular surrounding. Additionally, often the differentiation of the transplanted cells into a particular cell type largely depends on the nearby cells [22].

The differences between transplantation sites seem to be of great importance. Cells transplanted anteriorly presented better myelination than animals with cells transplanted posteriorly. Localization of transplant near the anterior parts of ventricles and at the same time close to corpus callosum in case of anterior transplantation might be favorable for induction of myelination. Glial progenitors transplanted in such an environment might be stimulated to differentiate into oligodendrocytes rather than remain as progenitors or become astrocytes and thus myelination is more pronounced. On the other hand, cGRPs transplanted posteriorly might migrate toward two important structures in terms of neurogenesis (hippocampus) or vital functions (brain stem). These are likely more essential structures from a therapeutic perspective than the corpus callosum. Myelination of the corpus callosum was negligible in a group of animals, which received more posterior cGRP transplantation, which is not surprising taking into account a distance between the transplantation site and this brain structure, but despite weaker myelination, the animals survived significantly longer. In our previous study we have shown that at the moment of death of control animals (~200 days), there was still no production of compact myelin by hGRPs [16]. Therefore, our current study further supports the hypothesis that myelination of the corpus callosum is probably not essential to extend survival of dysmyelinated mice and it might not be the best target for cell implantation in clinical translation as well as a biomarker of the outcome. Moreover, DiL tracing of the cells revealed that the posteriorly transplanted cells were mostly located on the same deepness level throughout the sections. The cells were clearly visible around lateral ventricles^,^ 3rd ventricle and the hippocampus, including striatum oriens and the corpus callosum area. What is interesting, within the anteriorly transplanted cells, if (at all) were possible to be visualized, the distribution does not differ significantly from posteriorly transplanted animals in terms of brain structure. However, it seems that this type of transplantation is less efficient in terms of cell presence, thus a lack of spectacular therapeutic effect might be the result of transplantation efficiency, whether due to the cell migration to the ventricular system or some other unknown factor enabling efficient transplantation and/or migration of the cells. The studies on optimal cell destination are certainly warranted, as we have previously shown that the survival of transplanted GRPs also strongly depends on their implantation site in the brain, although in this situation it was related to immune cell engagement [23]. More extensive migratory capacity of hGRPs was sufficient for colonization of brain stem area even after transplantation into the anterior aspect of lateral ventricle, whereas cGRPs do not have such a quality. It would be also interesting in the future to perform posterior transplantation of mGRPs (low migratory capacity) as transplanted at this location could be more therapeutic than previously transplanted anteriorly [16]. In order to exclude the presence of robust inflammation and/or edema, we have analyzed MR images. However, we were not able to notice any differences between A- and P-transplanted animals nor when compared with control. It might be due to the fact that responses such as edema or augmented inflammatory state might be visible rather in shorter time points (around 2 weeks and maximum 1 month after transplantation), whereas in this experiment we have performed MRI at the earliest time point, around 126 days post-transplantation.

The early assessment of cGRP biodistribution revealed another surprising result. We could not detect any cGRPs in a majority of anteriorly transplanted animals, whereas the same was true for only one mouse transplanted posteriorly. The reason for this apparent discrepancy might be that cGRPs transplanted to the larger CSF compartment such as the anterior lateral ventricle are less prone to adherence to the ventricular wall so can remain as a floating fraction and are washed out during cryo-processing. In contrast, cells injected into the smaller fluidic compartment due to posterior transplantation may facilitate firmer cell contact with the ventricular wall and persistence in the brain after tissue processing procedure. Moreover, we detected graft-derived myelin in the spinal cord of only one of the investigated mice, and it was anteriorly transplanted mice. One of the possible reasons for visualizing MBP in the spinal cord might be that intraventricularly transplanted GRPs traveled with cerebrospinal fluid (CSF) toward the spinal cord, settled down in a new niche and after differentiation became functional. While neonatally transplanted GRPs into the CSF migrate well into the brain parenchyma, cells injected at later stages of development are usually sediment and/or accumulate on the border of the brain or spinal cord parenchyma and demonstrate limited migration into the tissue [24,25]. We acknowledge the fact that this situation in applied conditions is rather unique and we cannot draw any particular conclusions; however, we intend to mark any atypical cell behavior.

The variability of cell distribution and homing is a major feature of all neonatal GRP transplantations, which translates to the inconsistency of therapeutic outcomes. Imaging of the transplantation procedure in real-time with tracking labeled cells might be a solution, as it was shown effective for guiding intra-arterial [26] and intraparenchymal transplantation [27]. Since we transplant cells to the fluid compartment, post mortem assessment of cell bio-distribution seems to be inadequate as the cells suspended in the fluid are lost during tissue processing. The majority of cell labeling studies use MRI of iron oxide-labeled cells; however, modalities based on nuclear medicine approaches are very compelling [28,29,30]. Radioactivity is quantitative in nature, it does not interfere with important imaging outcome measure techniques such as DTI or fMRI and is not subject to the numerous artifacts plaguing detection of iron oxide-labeled cells, which are especially pronounced in the spinal area.

Direct detection of transplanted canine cells is not possible due to the paucity of canine-specific antibodies, and this certainly is a limitation of our study. However, we were able to conclude on successful engraftment based on the functional effects of transplanted cells, namely myelin production. We have used two complementary methods: IHC detecting MBP, which is a protein, frequently preceding the process of myelination, and EM, which is a gold standard in detecting the ultrastructure of myelin. Importantly, no MBP staining or compact myelin beyond single axons was observed in non-transplanted mice, thus all positive findings can certainly be ascribed to transplanted cells. We can see the formation of compact myelin; however, not all of the axons were myelinated, and the number of myelin layers was lower in comparison with control wild-type animals. The diversity in migration patterns between mouse and human cells was also noticed by Windrem and colleagues [31]. Moreover, Klimczak et al., 2019 [32], compared the in vitro expression of migration marker PSA-NCAM between murine, canine and human GRPs, recognizing mGRPs as of the lowest level, cGRPs as moderate and hGRPs as highest. This indicates that canine GRPs may indeed demonstrate an intermediate migratory phenotype between those of murine and human origin. Besides, as shown by Kim and colleagues, intraventricularly transplanted GRPs in the experimental autoimmune encephalomyelitis (EAE) model were mostly localized in the ventricular system and were viable only until day 10 post-transplantation [33]. Since we have not observed robust myelination of corpus callosum by MRI in P-transplanted mice, we have not dedicated additional groups specifically for IHC and EM in these groups of animals.

Our study has shown that cGRPs appear to be functional and therapeutic in a mouse model of dysmyelination; however, there is more development needed ahead of the clinical translation of this approach in both human and veterinary medicine. It seems that migration of transplanted cGRPs is insufficient; therefore, the methods warranting better brain coverage are needed, such as multipoint transplantations, cell engineering to increase their migration or cell delivery approach supporting more widespread biodistribution such as intra-arterial injection. The ZEB1 transcription factor is a known regulator of cellular motility, and it has been shown to be responsible for increased migration of glioblastoma cells and neural stem cells; therefore, it is also a compelling strategy to enhance ZEB1 expression in GRPs [34,35,36]. We are also recently witnessing a therapeutic efficacy of induced pluripotent stem cell (iPS)-derived glial enriched progenitors, which may further facilitate interest in glial-based therapeutic approaches [37]. Obviously, each transplantation approach has its own challenges, such as the risk of hemorrhage due to multiple brain punctures, deterioration of therapeutic potential due to cell engineering or the need for cell diapedesis from arteries to the brain parenchyma. Cell labeling and imaging seem to be essential to guide future clinical application, as conclusive early assessment of cell bio-distribution using post-mortem techniques is obviously not possible.

## 5. Conclusions

The intraventricularly injected cGRPs engraft into the brain of dysmyelinated mice and appear to become functional after their transplantation. The survival benefit is independent of the myelination of the corpus callosum and depends on the transplantation location. Interestingly, the animals transplanted more posteriorly and in closer proximity to the brain stem lived significantly longer, whereas a positive effect was not observed after more anterior transplantation. In summary, there is more research needed on the GRP delivery prior to the clinical translation of this concept.

## Figures and Tables

**Table 1:** 139 mice used in the study and the animal allocation to experimental and control groups.

		Anteriorly Transplanted (shiv/rag2)	Posteriorly Transplanted (shiv/rag2)	Negative Control (shiv/rag2)	Positive Control (*Rag2* KO)
**Survival**		*N =* 38, MRI = 5	*N =* 12, MRI:5	*N =* 11	
IHC	18 weeks	*N =* 7(5)		*N =* 9, MRI:4	MRI:3
	>26 weeks	*N =* 7(3)			
EM	18 weeks	*N =* 5		28.5 weeks *N =* 5	28.5 weeks *N =* 5
	31 weeks	*N =* 5			
Bio-distribution	1 day	*N =* 6	*N =* 5		
	3 days	*N =* 5	*N =* 5		
	28 days	*N =* 7	*N =* 5	*N =* 2	

## Data Availability

The datasets used and/or analyzed during the current study are available from the corresponding author on reasonable request.

## Supplementary Materials

The following are available online at https://www.mdpi.com/article/10.3390/cells10112968/s1, Figure S1: Exemplary tail scans of whole-brain coronal slices demonstrating cerebral biodistribution of Dil-stained cGRPs transplanted anteriorly or posteriorly.
