# Supplementary material for: Myelin-Independent Therapeutic Potential of Canine Glial-Restricted Progenitors Transplanted in Mouse Model of Dysmyelinating Disease
Source: Cells. 2021 Nov 1;10(11):2968. doi: 10.3390/cells10112968 (PMC8616327; doi:10.3390/cells10112968)
Supplement: Supplementary file 1 [file cells-10-02968-s001.zip › cells-1359982-supplementary.pdf]

Supplementary Material

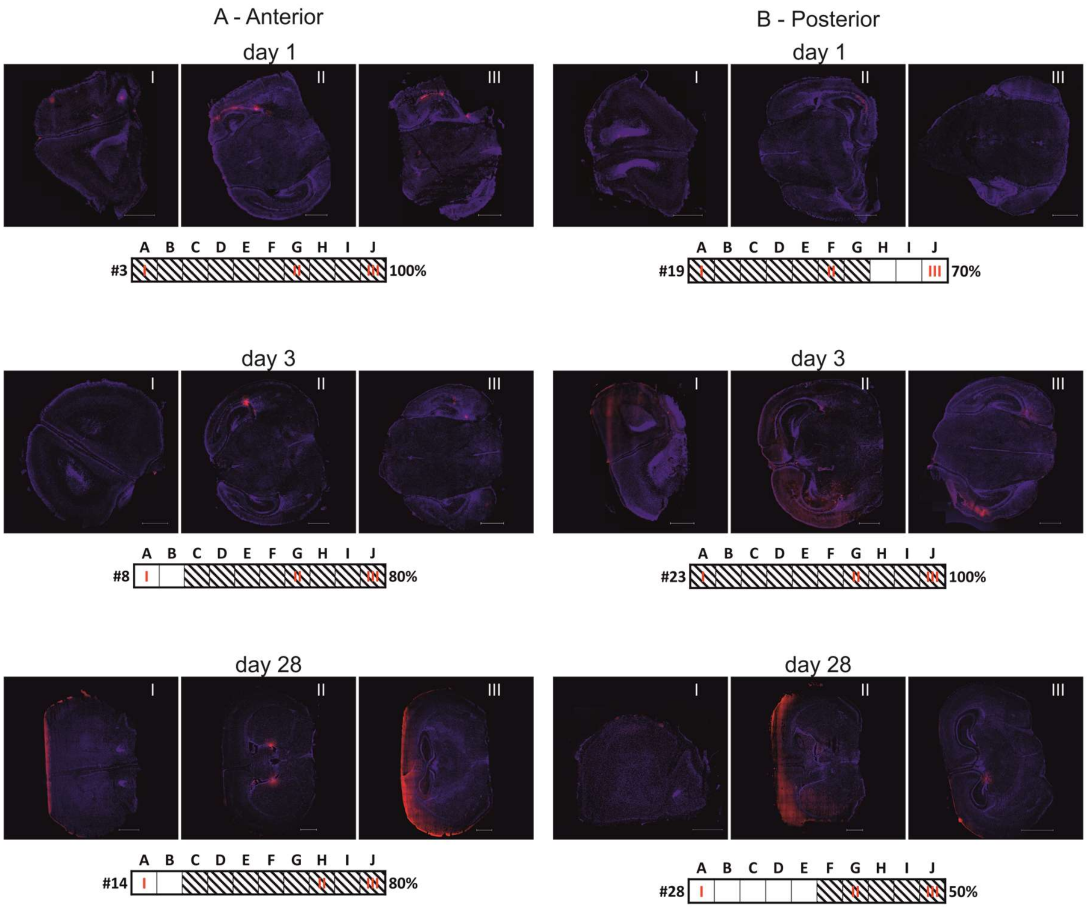

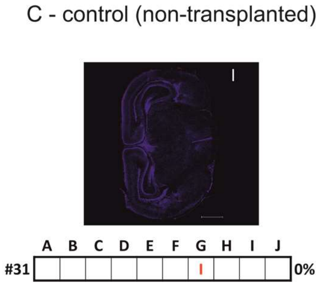

Supplementary Figure S1. Exemplary tail scans of whole-brain coronal slices demonstrating cerebral biodistribution of Dil-stained cGRPs transplanted anteriorly (A) or posteriorly (B) in shiverer mice. Slices were analysed 1, 3 or 28 days after transplantation. Each section within a row, indicated by letters A-J, reflects a 60 μm long brain area, with a 240 μm step between subsections. Patterned sections reflect Dil-positive areas, corresponding to cGRPs localization. Roman numerals (I-III) within sections reflect depth in the anterior-posterior axis at which each exemplary slice was collected. Percentage reflects overall Dil biodistribution across analysed brain slices from each exemplary mouse. Panel C represents non-transplanted (control) shiverer mouse. Each hashtag represents one animal. Nuclei were counterstained with Hoechst (blue). Scale bar: 1000 μm.
